# The embryo-derived protein PDI is highly conserved among placental mammals and alters the endometrial transcriptome and secretome *in vitro* across species with differing implantation strategies^†^

**DOI:** 10.1093/biolre/ioaf263

**Published:** 2025-12-04

**Authors:** Haidee Tinning, Alysha Taylor, Dapeng Wang, Anna Pullinger, Georgios Oikonomou, Miguel A Velazquez, Paul Thompson, Achim Treumann, Peter T Ruane, Mary J O’Connell, Niamh Forde

**Affiliations:** Discovery and Translational Sciences Department, Faculty of Medicine and Health, Leeds Institute of Cardiovascular and Metabolic Medicine, University of Leeds, Leeds, West Yorkshire, United Kingdom; Discovery and Translational Sciences Department, Faculty of Medicine and Health, Leeds Institute of Cardiovascular and Metabolic Medicine, University of Leeds, Leeds, West Yorkshire, United Kingdom; School of Biology, Faculty of Biological Sciences, University of Leeds, Leeds, West Yorkshire, United Kingdom; LeedsOmics, University of Leeds, Leeds, West Yorkshire, United Kingdom; Discovery and Translational Sciences Department, Faculty of Medicine and Health, Leeds Institute of Cardiovascular and Metabolic Medicine, University of Leeds, Leeds, West Yorkshire, United Kingdom; Department of Livestock and One Health, Institute of Infection, Veterinary and Ecological Sciences, University of Liverpool, Liverpool, Merseyside, United Kingdom; School of Natural and Environmental Sciences, Newcastle University, Newcastle upon Tyne, Tyne and Wear, United Kingdom; Protein and Proteome Analysis (NUPPA), Newcastle University, Newcastle upon Tyne, Tyne and Wear, United Kingdom; Protein and Proteome Analysis (NUPPA), Newcastle University, Newcastle upon Tyne, Tyne and Wear, United Kingdom; Faculty of Biology, Medicine and Health, Division of Developmental Biology and Medicine, Maternal and Fetal Health Research Centre, School of Medical Sciences, Saint Mary’s Hospital, Manchester Academic Health Sciences Centre, University of Manchester, Manchester, Greater Manchester, United Kingdom; School of Life Sciences, Faculty of Medicine and Health Sciences, University of Nottingham, Nottingham, Nottinghamshire, United Kingdom; Discovery and Translational Sciences Department, Faculty of Medicine and Health, Leeds Institute of Cardiovascular and Metabolic Medicine, University of Leeds, Leeds, West Yorkshire, United Kingdom; Centre for Reproductive Health, Institute for Regeneration and Repair, University of Edinburgh, Edinburgh, United Kingdom

**Keywords:** endometrium, bovine, secretome, transcriptome, microfluidics, conceptus-derived protein

## Abstract

Pregnancy establishment in mammals requires a complex sequence of events, including bi-lateral embryo-maternal communication, leading up to implantation. This is the time when most pregnancy loss occurs in mammals (including humans and food production species) and dysregulation in embryo-maternal communication contributes to pregnancy loss. Embryo-derived factors modify the function of the endometrium for pregnancy success. We hypothesise that these previously unexplored conceptus-derived proteins may be involved in altering the function of the endometrium to facilitate early pregnancy events in mammals with different early pregnancy phenotypes. Here, we show that protein disulphide-isomerase (PDI) is a highly conserved protein among mammals and provide evidence for a species-specific role for PDI in endometrial function in mammals with different implantation strategies. We show how PDI alters the endometrial transcriptome in human and bovine *in vitro* in a species-specific manner and using a microfluidic approach we demonstrate that it alters the secretome capability of the endometrium. We also provide evidence from *in vitro* assays using human-derived cells that *MNS1,* a transcript commonly downregulated in response to PDI in human and bovine endometrial epithelial cells, may be involved in the attachment phase of implantation. We propose that the trophoblast-derived protein PDI, is involved in supporting the modulation of the uterine luminal fluid (ULF) secreted by the endometrium to support conceptus nourishment, and in the process of embryo attachment to the uterine lumen for pregnancy success in mammals.

## Teaser

We provide evidence that a highly conserved protein (PDI) alters the endometrial transcriptome in a species- and cell-specific manner. Exposure of endometrial epithelia to PDI altered genes belonging to immune modulatory, pro-inflammatory, and adhesion-pathways. One transcript, *MNS1*, was commonly downregulated in endometrial epithelia from species with superficial (bovine) and invasive (human) implantation morphologies. Knockdown of *MNS1* expression in human endometrial epithelia altered the ability of human trophoblast BeWo spheroids to attach, suggesting a mechanism by which PDI affects implantation in human and bovine. In addition, using a microfluidics approach we have shown that PDI alters the secretome in a species-specific manner demonstrating PDI alters a key function of the endometrium in mammals. PDI alters the transcriptome and secretome of human and bovine endometrial cells *in vitro* in a species-specific manner.

## Graphical Abstract

**Graphical Abstract:**
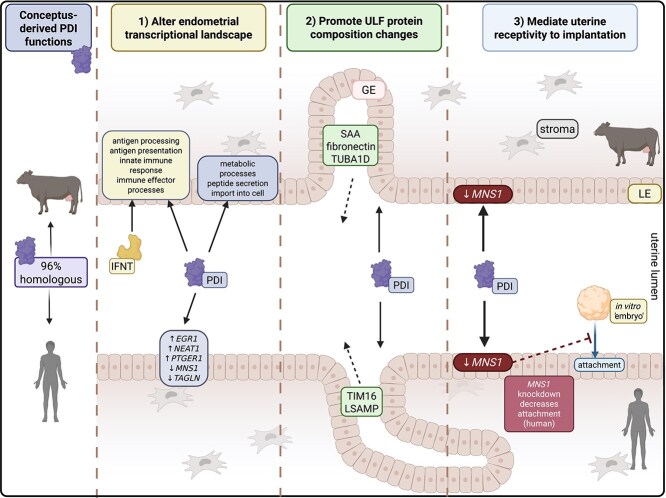

## Introduction

Placental mammals display a diversity in the timing, morphology, and molecular cues required for successful pregnancy [1]. Despite this diversity, the majority of pregnancy loss occurs in the pre- and peri-implantation stages of pregnancy in most mammalian species studied [2]. While some of these losses are attributable to defects in gametes and embryo development, transfer of competent embryos does not always result in pregnancy success. Dysregulation in signalling or communication between developing embryo and endometrium contributes to pregnancy loss.

The endometrium is a specialised heterogeneous tissue which lines the uterine cavity. Its main functions are to support growth and development of the embryo, respond to the pregnancy recognition signals from the embryo/conceptus, and facilitate receptivity to implantation. These functions are mediated by the different heterogeneous endometrial cells including fibroblast-like stromal cells, immune cells, a microvasculature system, and specialised epithelial cells. The endometrium also contains secretory glands which, along with the luminal epithelium, secrete the uterine luminal fluid (ULF). Secretion of the ULF is necessary to support growth and development of the embryo prior to the formation of the placenta [3]. The endometrium responds spatiotemporally to steroid hormones in the maternal circulation, including progesterone, concentrations of which rise and fall in a cyclic manner during the estrus or menstrual cycle. Progesterone-induced changes in the endometrium are essential for processes such as establishing receptivity to implantation. The ULF also changes in composition in response to progesterone [4, 5], and in response to signals from the embryo/conceptus [6].

Previous studies have determined that the transcriptomic response of the endometrium to the bovine conceptus during the pregnancy recognition period is greater than that of the pregnancy recognition signal alone [7]. To determine what other proteins may be involved in altering the endometrial transcriptome/ endometrial function, Day 16 bovine conceptuses were flushed from the uterine horn and cultured in medium in vitro to collect the conceptus conditioned medium which was subsequently subjected to quantitative proteomic analysis [8]. These proteins then represent conceptus-derived proteins which are secreted in vitro. Day 16 of pregnancy is associated with the maternal recognition of pregnancy (MRP) in cattle, a process essential for pregnancy establishment, mediated by the secretion of interferon tau (IFNT) from the conceptus which acts upon the endometrium. This is considered a critical timepoint which represents a key pregnancy checkpoint around which many early pregnancy losses occur [2]. Amongst the most abundant proteins identified in the conceptus conditioned medium was Protein disulphide-isomerase (PDI) (also known as protein disulphide-isomerase precursor or prolyl 4-hydroxylase -subunit [P4HB]) [8]. PDI is a 508 amino acid polypeptide belonging to the thioredoxin family of proteins and has multiple cellular functions, broadly involved in (1) redox regulation, (2) disulphide isomerase activity, (3) chaperone protein activity, and (4) redox-dependent chaperoning [9]. It is a highly abundant cellular protein, and has been found to be upregulated in different cancers [10] where it is often associated with poor outcomes [11], cancer progression, and tumour invasion [12]. PDI has also been linked with protein-misfolding associated neurodegenerative disorders, some cardiovascular disorders, and pathogen cellular entry in some infectious diseases such as HIV [13].

With several known functions, and the capacity to act as a sub-unit in multiple known complexes, PDI is involved in many cellular processes. In the endoplasmic reticulum, PDI is involved in the maintenance (forming and breaking) of disulphide bonds between cysteine residues during protein folding [14]. PDI can also stabilise and destabilise disulphide bonds in MHC class I molecules and is therefore involved in the ability of a cell to present antigens to the immune system [15]. PDI can also act as a chaperone protein for misfolded proteins in the endoplasmic reticulum [16]. PDI can also have other roles when it associates with other proteins as a complex, e.g., PDI is the β-subunit of microsomal triacylglycerol transfer protein (MTP) which is involved in incorporating triacylglycerols into lipoproteins. PDI can also act as the 2 β-subunits of prolyl 4-hydroxylase (P4H), which catalyses procollagen pro-α-chain proxyl hydroxylation in the endoplasmic reticulum [17].

More recently, it was proposed that PDI is also involved in endometrial receptivity to implantation in humans [18]. Fernando *et al* (2021) found that estrogen and progesterone altered the expression of certain PDI isoforms in different human endometrial epithelial cell lines [18]. Inhibition of PDI activity increased the attachment of Jeg-3 spheroids to AN3CA endometrial epithelial cells. Estrogen treatment has been shown to upregulate PDI expression in the endometrium of mice [19], and PDI expression is higher in the mid-secretory phase compared to the early-secretory phase endometrium of humans with unexplained infertility [20]. We have also shown that PDI alters expression of a core set of microRNAs in endometrial epithelia demonstrating its potential as a regulator of endometrial function [21]. PDI is a highly conserved conceptus-derived protein produced at the time of pregnancy recognition in cattle. We sought to determine if the role of PDI in implantation, including its effect on maternal endometrial tissues, is conserved in mammals with differing early pregnancy processes (human and bovine).

## Materials and methods

Unless otherwise stated all materials were sourced from Sigma-Aldrich. Tissue used in this study was obtained from a local abattoir from animals slaughtered for meat production, therefore no ethical approval was required.

### Conservation analysis of PDI

Analysis of species conservation was carried out as previously described [22]. Briefly, orthologs of PDI across 17 species from a range of placental mammals, non-placental mammals, and non-mammal outgroups were identified and extracted from Ensembl [23]. Multiple sequence alignment was performed using MAFFTv7.3 [24] under default settings, and using SeaView [25] for visualisation. The percentage identity matrix was generated in Clustal Omega [26].

### Recombinant PDI production

Recombinant bovine PDI (rbPDI) was produced at the Newcastle University Protein and Proteome Analysis centre using an *E. coli* pET3A expression vector system and subsequent purification steps, as described in [22]. Recombinant ovine IFNT (roIFNT) was kindly gifted by Professor Fuller Bazer for this study, produced in *P. pistoris* to >80% purity as described by Van Heeke *et al* [27]. All recombinant proteins were eluted/purified into PBS and stored at −80°C prior to use.

### Primary bovine endometrial cell culture

Primary bovine endometrial epithelial cells (bEECs) and primary bovine endometrial stromal cells (bESCs) were isolated by enzymatic digestion from late-luteal phase uterine tracts (n = 3) sourced from the local abattoir, as described previously [22]. bEECs and bESCs were maintained in complete bovine medium (RPMI 1640 [Gibco, Massachusetts, US], 10% charcoal-stripped FBS [PAA Cell Culture Company, UK], 1% antibiotic antimycotic solution [ABAM, Sigma-Aldrich, Missouri, USA]). bESCs at 150 000 cells per well (n = 5) and bEECs at 300 000 cells per well (n = 5) were plated into separate 6-well plates in 2 ml complete bovine medium. All cultures were maintained at 37°C/5% CO_2_ in a humidified incubator. At 70% confluence bEECs and bESCs were treated with one of the following for 24 hr: [1] Media control, [2] Vehicle control (PBS only), [3] 10 ng/ml rbPDI, [4] 100 ng/ml rbPDI, [5] 1000 ng/ml rbPDI or, [6] 1000 ng/ml rbPDI combined with 100 ng/ml roIFNT.

### Human endometrial cell culture

Ishikawa (ECACC 99040201, passage 10) immortalised human endometrial epithelial cells were plated in technical triplicate at 300,000 cells per well in a six well plate, in 2 ml complete human medium (DMEM/F12 [Gibco, Massachusetts, US], 10% FBS [PAA Cell Culture Company, UK], 1% Glutamine Streptomycin Penicillin [Gibco, Massachusetts, US]). The three replicates were from three different flasks of passage10 cells, which had been split from a single flask of passage 9 Ishikawa cells. All cultures were maintained at 37°C/5% CO_2_ in a humidified incubator. At 70% confluence Ishikawa cells were treated with one of the following for 24 h: [1] Media control, [2] Vehicle control (PBS only), [3] 10 ng/ml rbPDI, [4] 100 ng/ml rbPDI, or [5] 1000 ng/ml rbPDI.

### RNA extraction, cDNA conversion, and qRT-PCR

Following treatment, cells were washed with PBS, lysed in 400 μL *mir*Vana lysis solution from *mir*Vana RNA extraction kit (Invitrogen, St. Louis, US) and snap frozen. RNA was extracted from the lysed cell pellets using the MirVana RNA extraction kit (Invitrogen, St. Louis, US) as per manufacturer’s protocol. Genomic DNA removal was carried out using the DNA free kit (Thermo Fisher Scientific, Massachusetts, US) as per the manufacturer’s protocol. Two hundred ng/μl$\lceil $l RNA (bESCs and Ishikawa cells) or 50 ng/μl$\lceil $l RNA (bEECs) were reverse transcribed into cDNA using the High-Capacity cDNA Reverse Transcription Kit (Applied Biosystems, Massachusetts, US) as per the manufacturers protocol. Primers (IDT, Iowa, US) targeting bovine genes associated with early pregnancy gene expression changes in bovine endometrium and human genes associated with PDI (as determined by STRING DB analysis) were selected, including normaliser genes (bovine primers supplementary methods Table 1, human primers supplementary methods Table 2). Primers were designed using primer blast [28]. qRT-PCR was carried out on a Roche Lightcycler 480 II (Roche, Basel, Switzerland) as per the standard Roche protocol with an annealing temperature of 60°C. The 2^ΔΔCt^ method was applied [29], with *GAPDH* and *ACTB* used as normaliser genes. Normality of the ΔCt values was tested using the Shaprio-Wilk test (abnormally distributed *P <* 0.05) in Graphpad Prism. Significance was determined by ANOVA analysis on ΔCt values, ordinary one-way ANOVA with Dunnett multiple comparisons test was used for normally distributed datasets and Kruskal-Wallis test with Dunn multiple comparisons test was used for abnormally distributed datasets. Each condition was compared to the vehicle control.

**Table 1:** PDI percent amino acid identity matri*x*. The darker shade represents higher levels of PDI amino acid sequence similarity, and lighter shade indicates lower similarity between species

		*Human*	*Mouse Lemur*	*Bushbaby*	*Mouse*	*Sheep*	*Cow*	*Dolphin*	*Dog*	*Horse*	*Microbat*	*Elephant*	*Armadillo*	*Wallaby*	*Opossum*	*Platypus*	*Chicken*	*Zebrafish*
*Human*	ENSP00000327801	100	95	93	94	95	96	77	94	97	94	88	94	71	87	85	84	73
*Mouse Lemur*	ENSMICP00000001042	95	100	94	93	93	94	75	92	95	94	88	93	71	87	85	84	74
*Bushbaby*	ENSOGAP00000002121	93	94	100	92	92	92	74	91	93	92	86	91	71	87	83	83	72
*Mouse*	ENSMUSP00000026122	94	93	92	100	94	94	74	92	96	93	87	93	71	86	84	84	73
*Sheep*	ENSOARP00000019364	95	93	92	94	100	99	79	94	97	95	88	94	70	86	84	84	73
*Cow*	ENSBTAP00000007943	96	94	92	94	99	100	80	94	98	96	88	94	71	87	85	84	72
*Dolphin*	ENSTTRP00000011641	77	75	74	74	79	80	100	76	76	77	70	74	52	69	67	66	57
*Dog*	ENSCAFP00000008777	94	92	91	92	94	94	76	100	96	95	86	92	71	85	83	84	73
*Horse*	ENSECAP00000007958	97	95	93	96	97	98	76	96	100	97	94	94	72	91	86	87	75
*Microbat*	ENSMLUP00000017209	94	94	92	93	95	96	77	95	97	100	88	93	70	86	84	85	73
*Elephant*	ENSLAFP00000001175	88	88	86	87	88	88	70	86	94	88	100	90	67	83	81	83	69
*Armadillo*	ENSDNOP00000009104	94	93	91	93	94	94	74	92	94	93	90	100	70	88	85	84	73
*Wallaby*	ENSMEUP00000015288	71	71	71	71	70	71	52	71	72	70	67	70	100	78	70	66	58
*Opossum*	ENSMODP00000003645	87	87	87	86	86	87	69	85	91	86	83	88	78	100	82	80	70
*Platypus*	ENSOANP00000009770	85	85	83	84	84	85	67	83	86	84	81	85	70	82	100	79	71
*Chicken*	ENSGALP00000056764	84	84	83	84	84	84	66	84	87	85	83	84	66	80	79	100	70
*Zebrafish*	ENSDARP00000138657	73	74	72	73	73	72	57	73	75	73	69	73	58	70	71	70	100

**Table 2:** **Differentially abundant proteins present in conditioned medium following exposure of bovine endometrial epithelial cells to rbPDI.** Proteins identified by tandem mass spectrometry (FDR < 0.05), fold change abundance and p-value determined by students t-test compared to vehicle control samples

Accession ID	Protein name	Log2 Fold change abundance	*P*-value
A0A3Q1M3H8	Serum amyloid A protein OS=*Bos taurus* OX = 9913 GN=SAA3 PE = 3 SV = 1	3.721557	0.003687
F6Q9Q9	Protein disulfide-isomerase OS=*B. taurus* OX = 9913 GN=P4HB PE = 1 SV = 1	3.143024	8.27E-05
G5E5A9	Fibronectin OS=*B. taurus* OX = 9913 GN=FN1 PE = 4 SV = 2	1.315356	0.024178
Q2HJ86	Tubulin alpha-1D chain OS=*B. taurus* OX = 9913 GN = TUBA1D PE = 1 SV = 1	0.647515	0.038586
A0A3Q1LVC7	Ezrin OS=*B. taurus* OX = 9913 GN = EZR PE = 1 SV = 1	0.594263	0.045438
A0A3Q1MAJ8	Alpha-actinin-4 OS=*B. taurus* OX = 9913 GN = ACTN4 PE = 1 SV = 1	0.535577	0.048595
E1BEV7	Metalloendopeptidase OS=*B. taurus* OX = 9913 GN=BMP1 PE = 4 SV = 3	0.457946	0.011781
ENSEMBL:ENSBTAP00000016285	(*B. taurus*) similar to peptidoglycan recognition protein L [OS=*B. taurus*]	0.417109	0.007243
A0A3Q1MZC5	Calpain inhibitor OS=*B. taurus* OX = 9913 GN=CAST PE = 1 SV = 1	0.104419	0.024934
G3MZI7	Collagen type V alpha 1 chain OS=*B. taurus* OX = 9913 GN=COL5A1 PE = 4 SV = 2	−0.09254	0.018432
F6QND5	Fibrinogen alpha chain OS=*B. taurus* OX = 9913 GN=FGA PE = 4 SV = 1	−0.2967	0.019852
Q0VCM5	Inter-alpha-trypsin inhibitor heavy chain H1 OS=*B. taurus* OX = 9913 GN=ITIH1 PE = 1 SV = 1	−0.30207	0.025603
A0A3Q1MA31	Inter-alpha-trypsin inhibitor heavy chain H4 OS=*B. taurus* OX = 9913 GN=ITIH4 PE = 3 SV = 1	−0.39195	0.015608
P28800	Alpha-2-antiplasmin OS=*B. taurus* GN=SERPINF2 PE = 1 SV = 2	−0.45089	0.040426
A0A3Q1MGM5	Glutathione-independent PGD synthase OS=*B. taurus* OX = 9913 GN=PTGDS PE = 1 SV = 1	−0.51452	0.032937

### RNA sequencing and data analysis

Based on preliminary qRT-PCR data, the following samples were selected for RNA sequencing data analysis. bEEC and bESC (n = 3) samples: (1) Control, (2) Vehicle control, (3) 1000 ng/ml rbPDI, and (4) 100 ng/ml roIFNT +1000 ng/ml rbPDI. Ishikawa cells (n = 3) samples: (1) Control (2) Vehicle control, (3) 1000 ng/ml rbPDI. RNA libraries were sequenced at the NGS Facility at the University of Leeds where they were prepared and sequenced as per their standard protocols using the Illumina TruSeq Stranded Total kit according to manufacturer’s guidelines, as described in detail previously [22]. The RNA libraries were sequenced using the Illumina NextSeq 500 machine (Illumina, California, USA) with a single end 75 bp length read.

RNA sequencing data processing was conducted as described in detail in [22]. Data from the Ishikawa cells had 27.2 to 38.6 million raw reads per sample, with 64.0%–69.8% Subread successfully assigned fragments. Briefly, statistical test for differential gene expression was conducted via DESeq2 [30] with the cut-offs such as log2FoldChange >1 (or < −1) and padj <0.05. For principal component analysis (PCA) plotting of each group of samples, both protein-coding genes and long non-coding RNAs (lncRNAs) with RPKM value ≥1 in at least one sample were used and subsequently log2(RPKM+1) transformation and a quantile normalisation were applied. Overrepresentation enrichment analysis of differentially expressed protein-coding gene sets was executed using WebGestalt [31] for gene ontology terms and KEGG pathways. For gene ontology terms, biological process non-redundant datasets were chosen as functional database, and for both types of analyses, significance level was determined by FDR <0.05. Venn diagram analysis was performed using Venny [32].

### Attachment assays

Ishikawa cells (ECACC 99040201, passage 37, n = 5) were plated in 24-well plates (50,000 cells/well) in complete human medium. After 24 h incubation at 37°C /5% CO_2_, each well had the medium aspirated, 1 ml PBS (37°C) was added to each well to wash, and aspirated. To each well 400 μL of antibiotic-free human medium was then added. An siRNA solution was made up for each siRNA (1.4 μL 100uM siRNA [Horizon Discovery, UK] + 350 μL OptiMEM [Gibco, Massachusetts, US]) and a lipofectamine solution (3 μL Lipofectamine 2000 [Invitrogen, St. Louis, US] + 1.8 ml OptiMEM). Following a 20-min incubation, the following treatments were added to each corresponding well: (1) Control (100 μL OptiMEM only), (2) Vehicle control (50 μL OptiMEM +50 μL lipofectamine solution), (3) *MNS1* siGenome siRNA (50 μL siRNA solution +50 μL lipofectamine solution), (4) SiGenome non-targeting siRNA (50 μL siRNA solution +50 μL lipofectamine solution), (5) PBS vehicle control (20 μL PBS + 80 μL antibiotic-free human medium) and (6) PDI (1 μg/ml final concentration) (20 μL 25 μg/ml PDI stock +80 μL antibiotic-free human medium). Plates were then returned to the incubator (37°C/5% CO_2_) for 48 h before the assay.

24 h prior to the assay, a 6-well plate was coated with 1 ml of a 10% PVP (Sigma-Aldrich, Missouri, USA) solution for 30 min at room temperature. BeWo cells (ATCC CCL-98, passage 23) were lifted from a flask using trypsin (0.025%), pelleted at 500xg for 5 min, re-suspended in DMEM/F12 (Gibco, Massachusetts, US), and re-pelleted at 500xg for 5 min to remove all serum. The pellet was re-suspended in 3 ml DMEM/F12, cells counted on a haemocytometer, and diluted to 166,000 cells/ml in DMEM/F12. 3 ml cell suspension was added to each well after removing PVP solution and washing with 1 ml PBS. The plate was then placed on an orbital shaker at 60 rpm for 4 h in an incubator (37°C/5% CO_2_). Each well was then pipetted up and down 10 times and the plate placed back into the incubator overnight.

After a 12-h overnight incubation, BeWo spheroids were harvested by pipetting spheroids though a 100 μM strainer (Corning, New York, US) above a 40 μM strainer (Corning, New York, US) into a 50 ml falcon. The original BeWo culture dish was washed with DMEM/F12, and residual spheroids also passed through the strainers. The flow-through was discarded and the 40μ$\lceil $M strainer inverted over a 50 ml falcon and backwashed with 1 ml DMEM/F12.

Ishikawa cells after 48 h had medium aspirated, 500 μL DMEM/F12 added to remove traces of serum, aspirated, and 450 μL DMEM/F12 added. 50μ $\lceil $L BeWo spheroids were added to the well of Ishikawa cells containing 450 μL medium. The plate was then placed into an incubator (37°C/5% CO_2_) for 30 min to allow for attachment to occur. After 30 min, the wells were whole-well imaged on an EVOS light microscope (Thermo Fisher Scientific, Massachusetts, US), then media was carefully removed, 500 μL 10% neutral-buffered formalin (Epridia, Michigan, US) added gently and incubated at room temperature for 20 min to fix. After 20 min, the formalin was removed and 500 μL of PBS was added. The whole well was then imaged again using an EVOS microscope. Using QuPath software [33] each spheroid was counted in the before-incubation and the after-incubation images for each well. Spheroids in the dark well rim were not counted, and clumps of spheroids had their number estimated. The % of attachment (spheroids remaining following fixing compared to number added to well) was calculated. A Shapiro–Wilk test was used to identify distribution normality for both datasets in Graphpad Prism. For the rbPDI dataset, an unpaired t-test with Welch correction was used to determine significance. For the *MNS1* siRNA dataset, an ordinary one-way ANOVA analysis with Tukey multiple comparisons test was used to determine significance. Graphpad Prism was used to perform statistical testing.

### 2D microfluidics

**Figure 1:**
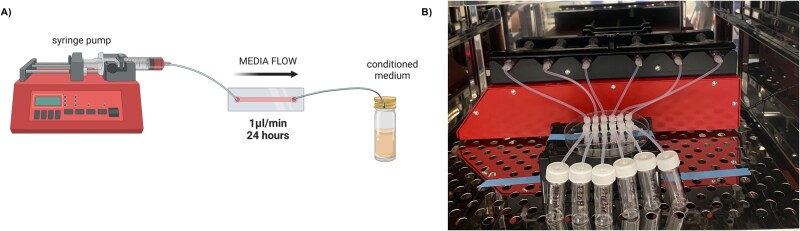
(A) Graphical representation of microfluidics experimental setup. Syringe pump holds syringe filled with medium containing treatment is connected via tubing and connectors to a microfluidic device to flow medium through the channel (set flow rate 1$\lceil $Lμl/min). (B) Representative example of a 6-channel Ibidi device attached to a syringe pump in an incubator.

Full detail on the microfluidic device seeding, set up, treatments, culture, conditioned medium collection, and proteomic analysis is contained in the supplementary methods. Briefly, 2D microfluidic devices (IbiFlow 0.4 channel slides Ibidi, Germany) were seeded with human endometrial epithelial Ishikawa cells (n = 3) or bovine endometrial epithelial primary cells (n = 3). Devices were attached to syringes containing either vehicle control or rbPDI (100 μg/ml) and treatment occurred for 24 h (as seen in Figure 1). Conditioned medium was collected from the outlet and subjected to tandem mass tag mass spectrometry analysis (Bristol Proteomics Facility). The raw output files were processed and quantified using Proteome Discoverer software v2.1 (Thermo Scientific) and searched against the UniProt *Bos taurus* or *Homo sapiens* databases. The reverse database search option was enabled, and all data was filtered to satisfy false discovery rate (FDR) of 5%.

The resulting list of proteins for each sample were then analysed as described by Aguilan *et al* to determine the fold change in protein abundance between conditioned medium from rbPDI treated cells compared to vehicle control (PBS only) and the associated p-value [34]*.* First, any proteins which were present in the list but had no abundance values for any sample tested were removed from the list. Next, the data was transformed to a normal distribution using a log_2_ calculation followed by normalisation of the data by scaling each value against the average of all proteins within each sample. Next, the correlation slope of the average column for all samples compared with that of each sample was calculated. Then the log_2_ for each protein abundance was divided by the correlation slope for the sample, to further normalise the data by slope. Following this 2-step normalisation, any blank cells were randomly assigned a value which fits within the distribution (called deterministic minimum imputation) between 0 and 0.3. From the assigned value 2.5X the standard deviation of all values from that sample was removed. Fold changes were then calculated by taking the average of the vehicle control samples from the average of the rbPDI treated conditioned medium samples. Therefore, those proteins with a positive fold change are more abundant in the rbPDI conditioned medium samples compared to vehicle control, and the proteins with a negative fold change value are less abundant. P-value significance was calculated by paired two-tailed t-test, all comparisons with a p-value <0.05 were considered significantly different.

## Results

### PDI is a highly conserved protein across mammals

The PDI amino acid sequence is highly conserved across representative placental mammals (>80% amino acid sequence conservation), and 96% conserved between human and bovine (Table 1). Specifically, this study used the conceptus-derived protein identified by Forde *et al* (2015) with identifiers gi|27806501 and A6H7J6_BOVIN, which corresponds to P0530 (PDIA1_BOVIN) in the UniProtKB reviewed database (Swiss-Prot). This protein is now named protein disulphide isomerase, gene symbol *P4HB*, a 510 amino acid protein. ENSBTAP00000007943 is the Ensembl protein identifier which corresponds to this protein and was used to compare the amino acid sequence to that of other species (as seen in Table 1), including human (PDIA_Human in the UniProt database P07237).

### Recombinant bovine PDI alters specific transcripts in bESCs and human endometrial Ishikawa cells

**Figure 2:**
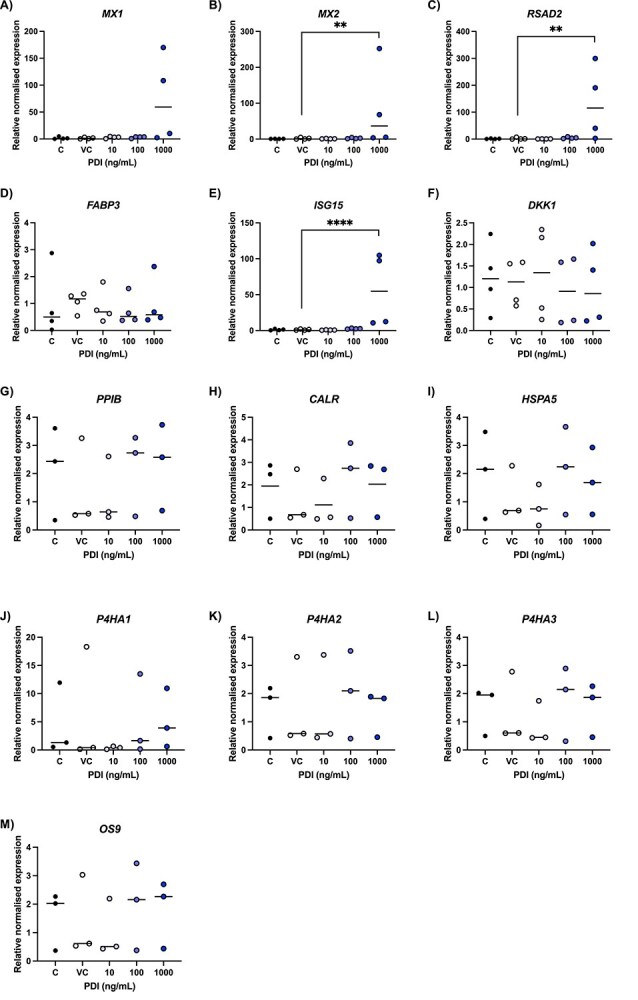
(A–F) Relative expression of interferon-stimulated genes in bovine endometrial stromal cells following treatment with rbPDI (10/100/1000 ng/ml) for 24 h (n = 4), alongside media control (C) and vehicle control (VC) conditions. Relative gene expression to the vehicle control samples determined by qRT-PCR and the 2^-ΔΔCt method, with *ACTB* and *GAPDH* as normaliser genes. Normality determined using the Shaprio-Wilk test (*P* < 0.05). Significance determined by ANOVA analysis, ordinary one-way ANOVA with Dunnett multiple comparisons test for normally distributed datasets and Kruskal-Wallis test with Dunn multiple comparisons test for abnormally distributed datasets. Each condition was compared to the VC. ^*^P < 0.05.. Figure created in Graphpad Prism. Line at median. (G–M) Relative expression of PDI-associated genes in human Ishikawa endometrial epithelial cells following treatment with rbPDI (10/100/1000 ng/ml) for 24 h (n = 3), alongside media control (C) and vehicle control (VC) conditions. Relative gene expression to the vehicle control samples determined by qRT-PCR and the 2^-ΔΔCt method, with *ACTB* and *GAPDH* as normaliser genes. Normality determined using the Shapiro–Wilk test (*P* < 0.05) on ΔCt values. Significance determined by ANOVA analysis on ΔCt values, ordinary one-way ANOVA with Dunnett multiple comparisons test for normally distributed datasets and Kruskal-Wallis test with Dunn multiple comparisons test for abnormally distributed datasets. Each condition was compared to the VC. ^*^P < 0.05, ^*^^*^P < 0.01, ^*^^*^^*^P < 0.001, ^*^^*^^*^^*^P < 0.0001. Figure created in Graphpad Prism on 2^-ΔΔCt values for visualisation of normalised relative expression. Line at median.

When treated with rbPDI at the highest concentration used (1000 ng/ml rbPDI), there was a significant increase in the expression of bovine early-pregnancy associated transcripts *MX2*, *RSAD2,* and *ISG15* (*P* < 0.05), but not *MX1*, *FABP3*, or *DKK1*, in bESCs compared to vehicle control samples (Figure 2A–F).

As seen in Figure 2G–M), rbPDI treatment of Ishikawa cells did not significantly alter the expression of any of the transcripts assessed (*PPIB, CALR, HSPA5, OS9, P4HA1, P4HA2, P4HA3*) at the concentrations (10/100/1000 ng/ml) and treatment duration (24 h) tested compared to vehicle control samples. These transcripts were selected as they encode proteins which were shown through STRING-DB analysis to interact or be associated with PDI.

### Recombinant bovine PDI induces a transcriptional response in bEEC and bESCs

**Figure 3:**
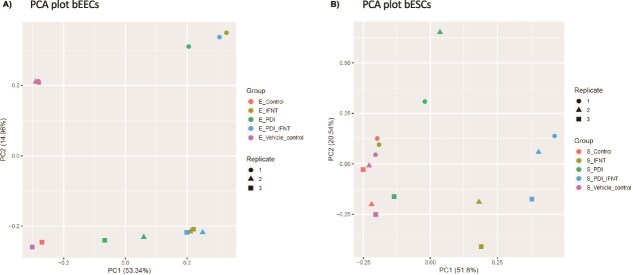
Principal component analysis describing the transcriptional effect of rbPDI and roIFT. Including rbPDI treatment alone, roIFNT treatment alone, and both in combination compared to control and vehicle control samples in (A) bovine endometrial epithelial cells or (B) bovine endometrial stromal cells (n = 3). Data points indicate each replicate and data point colours indicate treatment groups. Control samples (_Control), vehicle control samples (_vehicle_control), roIFNT treatment (_IFNT), rbPDI treatment (_PDI), and rbPDI + roIFNT in combination (_PDI_IFNT).

Based on the qRT-PCR data demonstrating that the transcriptional response to rbPDI in bESCs occurred in response to the highest concentration of rbPDI tested (1000 ng/ml), the samples treated with 1000 ng/ml rbPDI were used for RNA sequencing. rbPDI induced a transcriptional response in both bESCs and bEECs distinct from controls, as seen in Figure 3. The PCA plots show that the control samples separate from the samples treated with rbPDI in PC1 (Figure 3A and B). In bEECs, there was no visual distinction between the roIFNT and the rbPDI combined with roIFNT samples in PC1 or PC2 (Figure 3A). However, in bESCs, there was a distinction between rbPDI alone, roIFNT alone, and roIFNT in combination with rbPDI, in PC1 (Figure 3B). Specifically, rbPDI alone treated samples were closest to controls in PC1, with roIFNT alone being further from controls in PC1, and roIFNT in combination with rbPDI being furthest from controls in PC1, indicating a potentially additive effect which is explored in the next section. rbPDI treatment alone led to a large variation in samples across PC2, whereas rbPDI in combination with roIFNT reduced this variation in PC2.

**Figure 4:**
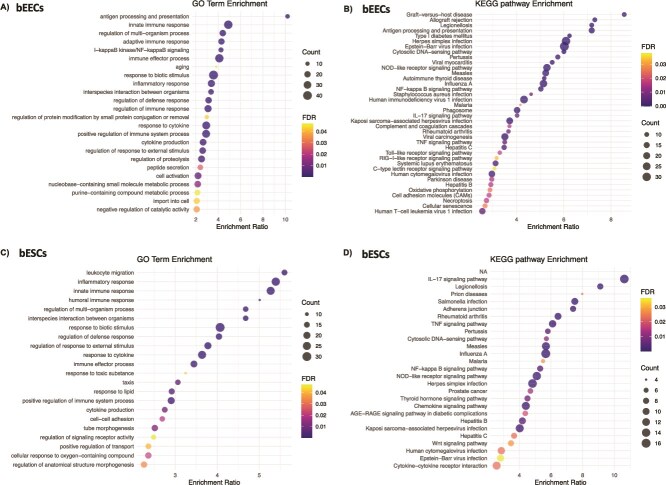
(A) Enriched GO terms and (B) KEGG pathways in DEGs specific to bEECs and (C) GO terms and (D) KEGG pathways in DEGs specific to bESCs treated with rbPDI. DEGs including uncharacterised transcripts compared to vehicle control (VC) samples (padj<0.05) analysed by WebGestalt to determine enriched GO terms/KEGG pathways (FDR <0.05). Full data in supplementary tables 2, 3, 5, 6.

rbPDI treatment altered the expression of 448 transcripts compared to vehicle control in bEECs, including four upregulated lncRNAs (Supplementary table S1). Of the protein coding transcripts, 365 were upregulated and 83 downregulated compared to vehicle control. Twenty-four GO terms (Figure 4A, Supplementary Tables S2) and 38 KEGG pathways were enriched within the DEGs compared to vehicle control (Figure 4B, Supplementary table S3). GO enrichment analysis revealed overrepresentation of terms relating to “antigen processing and presentation” (GO:0019882), “innate immune response” (GO:0045087), “regulation of multi-organism process” (GO:0043900), “adaptive immune response” (GO:0002250), peptide secretion (GO:0002790), import into cell (GO:0098657), “signal transduction in absence of ligand” (GO:0055086), and “purine-containing compound metabolic process” (GO:0072521). KEGG pathway analysis demonstrated enrichment for “graft-versus-host disease” (bta05332), “allograft rejection” (bta05330), “antigen processing and presentation” (bta04612), and “cell-adhesion molecules” (bta04514).

In bESCs, rbPDI treatment altered the expression of 306 DEGs compared to vehicle control, all of which were protein coding transcripts (Supplementary table S4). Three hundred protein coding transcripts were upregulated and six downregulated compared to vehicle control. Twenty-two GO terms (Figure 4C, Supplementary table S5) and 26 KEGG pathways were enriched with the DEGs compared to vehicle control (Figure 4D, Supplementary table S6). Enriched GO terms included “leukocyte migration” (GO:0050900), “inflammatory response” (GO:0006954), “innate immune response” (GO:0045087), “humoral immune response” (GO:0006959), and “regulation of multi-organism process” (GO:0043900), and enriched KEGG pathways included “IL-17 signaling pathway” (bta04657), “legionellosis” (bta05134), and “prion diseases” (bta05020).

**Figure 5:**
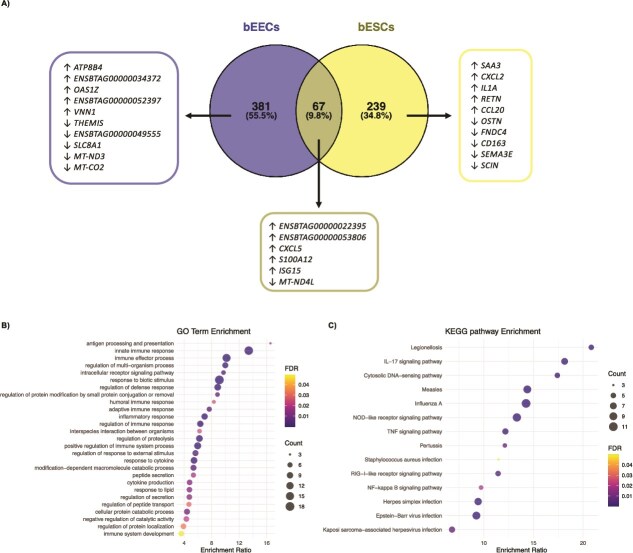
Comparison of DEGs induced by rbPDI in bEECs and bESCs and enriched GO terms and KEGG pathways associated. (A) Venn diagram analysis of rbPDI treatment induced DEGs in bEECs vs bESCs. rbPDI compared to vehicle control samples (n = 3) in bovine endometrial epithelial cells (bEECs) and stromal cells (bESCs). Top 5 up or downregulated transcripts in each group included. Full data Supplementary table 7. (B) Enriched GO terms and (C) KEGG pathways in 67 DEGs specific to both bEECs and bESCs treated with rbPDI. DEGs including uncharacterised transcripts compared to vehicle control (VC) samples (padj<0.05) analysed by WebGestalt to determine enriched go terms/KEGG pathways (FDR <0.05). Full data in Supplementary tables 8 & 9.

Venn diagram analysis of the DEGs induced by rbPDI in bEECs and bESCs compared to vehicle control samples demonstrated the transcriptional response induced by rbPDI broadly differed between bEECs and bESCs (Figure 5A, Supplementary table S7), although 67 transcripts were commonly altered in both cell types. Downstream analysis on the 67 transcripts revealed 27 overrepresented GO terms (Figure 5B, Supplementary table S8), and 14 enriched KEGG pathways (Figure 5C, Supplementary table S9). Enriched GO terms included “antigen processing and presentation” (GO:0019882), “innate immune response” (GO:0045087), “immune effector process” (GO:0002252), and “regulation of multi-organism process (GO:0043900). Enriched KEGG pathways included ‘legionellosis’ (bta05134), ‘IL-17 signaling pathway” (bta04657), and “cytosolic DNA-sensing pathway” (bta04623).

### Recombinant bovine PDI alone, and in combination with roIFNT, induces a distinct transcriptional response to roIFNT alone in bESC and bEECs

bEECs were treated with rbPDI in combination with roIFNT to examine the effect of treatment alongside the well characterised conceptus-derived MRP signal IFNT which would be present in large quantities on day 16 of pregnancy. bEECs treated with roIFNT in combination with rbPDI led to 1415 DEGs compared to vehicle control samples. Of the 1385 altered protein coding transcripts, 974 were upregulated and 411 downregulated compared to vehicle control. Of the altered 30 lncRNAs, 23 were upregulated and seven downregulated compared to vehicle control (Supplementary table S10). Forty-three GO terms and 36 KEGG pathways were enriched among the DEGs compared to vehicle control (Supplementary table S11 + S12). Enriched GO terms included “antigen processing and presentation” (GO:0019882), “cellular modified amino acid biosynthetic process” (GO:0042398), “necrotic cell death” (GO:0070265), “fatty acid derived metabolic process” (GO:1901568), and “NIK/NF-kappaB signaling” (GO:0038061). Enriched KEGG pathways included “Glycosaminoglycan biosynthesis” (bta00533), “Pantothenate and CoA biosynthesis” (bta00770), and “Nicotinate and nicotinamide metabolism” (bta00760).

**Figure 6:**
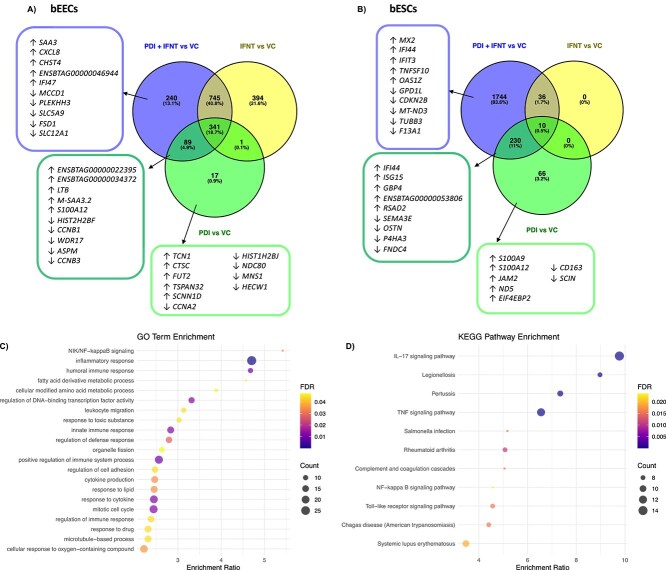
Venn diagram analysis of DEGs induced in bovine endometrial (A) epithelial or (B) stromal cells treated with roIFNT (from Tinning *et al* 2020), rbPDI, or rbPDI in combination with roIFNT when compared to vehicle control (VC) samples. Full data in Supplementary tables 13 & 21. Enriched (C) GO terms and (D) KEGG pathways in DEGs specific to bEECs treated with rbPDI vs VC or rbPDI + roIFNT, but not induced by roIFNT, vs VC. DEGs including uncharacterised transcripts compared to VC samples (padj<0.05) analysed by WebGestalt to determine enriched go terms (FDR <0.05), full data in supplementary tables 16 & 17.

Venn diagram analysis (Figure 6A, Supplementary table S13) determined that 341 transcripts were commonly differentially expressed in bEECs in response to all three treatments of: roIFNT only [22], rbPDI only, and rbPDI and roIFNT in combination when compared to vehicle control samples. rbPDI and roIFNT in combination elicited the differential expression of 240 transcripts which were not altered in roIFNT or rbPDI only treatments. Eighty-nine transcripts were differentially expressed in response to PDI alone and in combination with roIFNT, but not by roIFNT alone. Additionally, 17 transcripts were uniquely differentially expressed in response to rbPDI, but not when treated in combination with roIFNT. Further analysis of the 341 DEGs commonly altered by both rbPDI and roIFNT alone and in combination in bEECs compared to vehicle control revealed 13 enriched GO terms and 23 enriched KEGG pathways (Supplementary table S14 + S15). Enriched GO terms included “antigen processing and presentation” (GO:0019882), “aging” (GO:0007568), “innate immune response” (GO:0045087), “regulation of multi-organism process” (GO:0043900), and “immune effector process” (GO:0002252). Enriched KEGG pathways included “antigen processing and presentation” (bta04612), “graft-versus-host disease” (bta05332), and “allograft rejection” (bta05330). Further analysis of the 346 DEGs (Supplementary Table S16) commonly altered by both rbPDI alone and rbPDI in combination with roIFNT, but not roIFNT alone in bEECs compared to vehicle control revealed 21 enriched GO terms (Figure 6C, Supplementary Table S17) and 11 enriched KEGG pathways (Figure 6D, Supplementary Table S17). Enriched GO terms included “NIK/NF-kappaB signaling” (GO:0038061), “inflammatory response” (GO:0006954), “humoral immune response” (GO:0006959), “fatty acid derivative metabolic process” (GO:1901568), “cellular modified amino acid metabolic process” (GO:0006575), and “regulation of cell adhesion” (GO:0030155). Enriched KEGG pathways included “IL-17 signaling pathway” (bta04657), “legionellosis” (bta05134), “pertussis” (bta05133), “TNF signaling pathway” (bta04668), “NF-kappaB sigaling pathway” (bta04064), and “toll-like receptor signaling pathway” (bta04620).

bESCs treated with rbPDI together with roIFNT elicited the differential expression of 2020 transcripts, including 32 lncRNAs and 1988 protein-coding transcripts, compared to vehicle control samples. Of the lncRNAs, 20 were upregulated and 12 downregulated compared to vehicle control, and of the protein-coding transcripts, 1367 were upregulated and 621 downregulated compared to vehicle control (Supplementary Table S18). Fifty-three GO terms and 62 KEGG pathways were enriched in the DEGs compared to vehicle control (Supplementary table S19 + S20). Enriched GO terms included “antigen processing and presentation” (GO:0019882), “necrotic cell death” (GO:0070265), “leukocyte apoptotic process” (GO:0071887), “leukocyte apoptotic process” (GO:0071887), and “signal transduction in absence of ligand” (GO:0038034). Enriched KEGG pathways included “steroid biosynthesis” (bta00100), “sulfur metabolism” (bta00920), and “legionellosis” (bta05134).

In bESCs, a comparison of roIFNT and rbPDI treatment in bESCs revealed that of the 44 DEGs altered by roIFNT and the 306 DEGs altered by rbPDI compared to vehicle control 8 transcripts were commonly altered by both treatments alone and in combination (Figure 6B, Supplementary Table S19). Venn analysis showed that the 44 transcripts altered by roIFNT treatment were also altered by roIFNT treatment in combination with rbPDI. Eight transcripts were commonly altered by all three treatment groups, and 232 of the transcripts altered by rbPDI alone were also altered when treated with rbPDI in combination with roIFNT. Sixty-six transcripts were uniquely altered by PDI treatment alone, but not in combination with roIFNT or roIFNT alone. Further analysis on the eight DEGs altered in response to all three treatments did not result in any significantly enriched GO terms or KEGG pathways.

### Human endometrial epithelial Ishikawa cells have a limited transcriptional response to rbPDI

**Figure 7:**
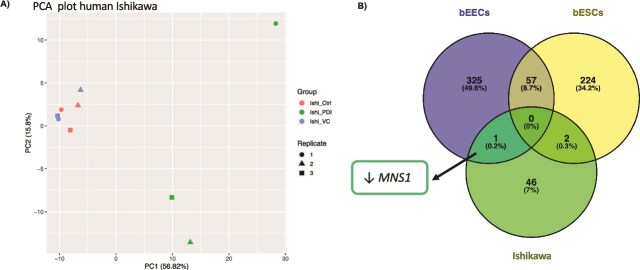
(A) Principal component analysis describing the transcriptional response to rbPDI treatment in human endometrial Ishikawa cells. Control samples (_Ctrl), vehicle control samples (_VC), and rbPDI (_PDI). (B) Venn diagram analysis of rbPDI induced DEGs. rbPDI compared to vehicle controls samples (n = 3) in human endometrial epithelial Ishikawa cells (hEECs), bovine endometrial epithelial cells (bEECs), and bovine endometrial stromal cells (bESCs). Includes characterised transcripts only. Full data in Supplementary table 23.

rbPDI elicited a transcriptional response in human endometrial epithelial Ishikawa cells *in vitro* compared to vehicle control treatment, based on the PCA plot (Figure 7A). The rbPDI treated samples clustered separately to the controls in PC1. Forty-nine transcripts (7 of which are lncRNAs) were altered by rbPDI compared to vehicle control (Supplementary Table S20). Twenty-six of the protein-coding transcripts were upregulated and 16 downregulated compared to vehicle control, including *EGR1* (encoding early growth response protein 1), *NEAT1* (nuclear paraspeckle assembly transcript 1) lncRNA, and *PTGER1* (encoding prostaglandin E2 receptor 1) which were increased in expression, and *MNS1* (encoding meiosis-specific nuclear structure 1 protein) and *TAGLN* (encoding trangelin protein) which were decreased in expression compared to vehicle controls. No significantly enriched GO terms or KEGG pathways were identified. Very few transcripts were conserved in the transcriptional response to rbPDI between bovine and human compared to vehicle control. However, there was a shared response to rbPDI in both bovine and human endometrial epithelial cells. A Venn comparison of DEGs altered by rbPDI treatment compared to vehicle control samples in bEECs, bESCs, and Ishikawa cells demonstrated that of the 49 characterised transcripts altered in Ishikawa cells, 1 transcript (*MNS1)* was also altered in bEECs, and two transcripts (*NLGN2-* encoding neuroligin-2 and *ULK1-* encoding unc-51 like autophagy activating kinase 1) were also altered in bESCs (Figure 7B, Supplementary Table S21). As we saw a limited transcriptional response to rbPDI in Ishikawa cells, and any conceptus-derived factors would first encounter the endometrial epithelial cells, we did not extend this project to include human endometrial stromal cells.

### *MNS1* knockdown alters *in vitro* trophoblast spheroid attachment to human endometrial epithelial Ishikawa cells

**Figure 8:**
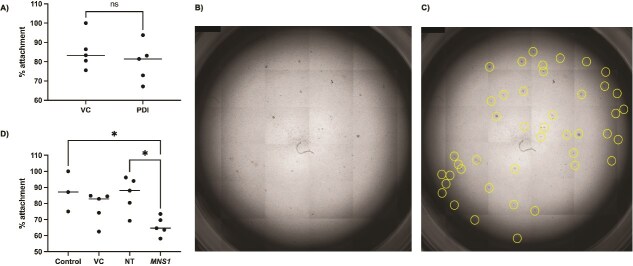
rbPDI and *MNS1* knockdown effect upon trophoblast spheroid attachment to endometrial cells. Endometrial epithelial human Ishikawa cells treated with (A) rbPDI (PDI) or vehicle control (VC), or (D) control, lipofectamine only vehicle control (VC), non-targeting siRNA (NT), or siRNA targeting *MNS1* (*MNS1*), were co-cultured with BeWo human trophoblast spheroids to investigate how treatment alters the percentage of spheroids adhering to the endometrial cells (n = 5). A Shapiro–Wilk test demonstrated normal distribution for both datasets (*P* > 0.05), and (A) unpaired t-test with Welch’s correction or (D) ordinary one-way ANOVA analysis with Tukey multiple comparisons test was used in Graphpad Prism to determine significance. Graphs created in Graphpad Prism. * = *P* < 0.05. All other comparisons in (D) were non-significant (*P* > 0.05). The line represents the median. (B and C) Representative image of the spheroid attachment assay, plus counting of spheroids in QuPath (yellow circles indicate spheroids). Image taken on an EVOS microscope.

Human endometrial cells pre-treated with rbPDI for 48 h had no significant difference in BeWo spheroid attachment to human endometrial epithelial Ishikawa cells compared to those pre-treated with vehicle control at the concentration tested (1000 ng/ml) (Figure 8A–C). *MNS1* was commonly downregulated in both Ishikawa human endometrial epithelial and bEECs in response to rbPDI treatment (Figure 7B). *MNS1* knockdown was quantified to ensure knockdown of expression to >80%. qRT-PCR demonstrated that *MNS1* was knocked down by 86.1% compared to non-targeting siRNA (Supplementary methods Fig. 1). Human endometrial cells pre-treated with siRNA targeting *MNS1* under optimised conditions for 48 h significantly reduced BeWo spheroid attachment to Ishikawa cells when compared to those pre-treated with a non-targeting siRNA (Figure 8D).

### Using a microfluidic device, exposure of endometrial epithelial cells to PDI alters the endometrial secretome in a species-specific manner

Two human proteins were identified as differentially abundant within conditioned medium between vehicle control and rbPDI treated Ishikawa cells in microfluidic devices (*P* < 0.05), specifically mitochondrial import inner membrane translocase subunit TIM16 (gene name *PAM16,* log2 fold change 0.16) and limbic system-associated membrane protein (gene name *LSAMP*, log2 fold change 0.08). As expected, the protein with the highest fold change is bovine PDI, which was added to the medium.

Fifteen proteins were identified as differentially abundant within the conditioned medium between vehicle control and rbPDI treated bEECs in microfluidic devices (*P* < 0.05) (Table 2). As expected, bovine PDI was present in the list of differentially expressed genes as it was added to the culture medium. Aside from PDI, eight proteins: serum amyloid A, fibronectin, tubulin alpha 1D chain, ezrin, alpha-actinin-4, metalloendopeptidase, similar to peptidoglycan recognition protein, and calpain inhibitor were increased in abundance compared to vehicle control, and six proteins: collagen type V alpha 1 chain, fibrinogen alpha chain, inter-alpha-trypsin inhibitor heavy chain H1, inter-alpha-trypsin inhibitor heavy chain H4, alpha-2-antiplasmin, and glutathione-independent PGD synthase decreased in abundance compared to vehicle control. Interestingly, serum amyloid A protein was even more highly differentially abundant compared to vehicle control than rbPDI (which was added artificially at a relatively high concentration).

### Discussion

We have shown that the PDI protein sequence is highly conserved across placental mammals, with species-specific influence on the endometrium, with rbPDI inducing a significant transcriptional response in bovine and human endometrial cells *in vitro*. Many of the transcripts induced by rbPDI are also induced by roIFNT *in vitro.* The response is cell specific in the bovine, while in human epithelial cells rbPDI induces a limited transcriptional response. One transcript (*MNS1*) was commonly downregulated in both bovine and human endometrial epithelial cells and knockdown of this transcript altered the ability of trophoblast spheroids to attach. rbPDI treatment of bEECs and Ishikawa cells under flow in a microfluidic device revealed that rbPDI influences the secretome of endometrial epithelial cells in a species-specific manner.

### PDI secretion supports the transcriptional response to IFNT in the bovine endometrium

The transcriptional response to PDI mostly overlaps with the transcriptional response *in vitro* to roIFNT. Of those DEGs commonly altered by rbPDI and roIFNT when treated separately and in combination when compared to vehicle control, the GO terms antigen processing and presentation, innate immune response, and immune effector processes are amongst the highest enriched. These data support the hypothesis that PDI may be involved in the interferon response and/or immune response induced by IFNT. Therefore, this previously uncharacterized embryo-derived protein, PDI, may be secreted by the bovine conceptus to support the actions of IFNT to modulate the endometrial transcriptome or mediate the immune response to IFNT. PDI has previously been linked to immune regulation and could induce tolerogenic dendritic immune cells [35], although the mechanism was not elucidated. We hypothesise that PDI may support the immune modulation effects of IFNT in the bovine endometrium, and may act to tolerise the maternal immune response to the semi-allographic conceptus [36]. The KEGG pathways “allograft rejection” and “antigen processing and presentation” were enriched when treated with rbPDI compared to vehicle control, further supporting this hypothesis.

### PDI elicits a unique transcriptional response to IFNT in the bovine endometrium

These data provide further evidence that not all of the transcriptional effects seen within the bovine endometrium *in vivo* in response to the day 15/18 conceptus are due to interferon stimulation [7, 37]. Our data supports the hypothesis that the conceptus-derived protein PDI, may be responsible for some of these observed differences [8]. As described, there were several DEGs elicited in response to rbPDI alone or in combination with roIFNT, that were not altered by roIFNT alone. This indicates that PDI elicits a unique transcriptional response in the endometrium which may support early pregnancy processes. The transcriptional response to rbPDI in bEECs included multiple enriched GO terms involved in metabolic processes, peptide secretion, and import into cell, which are not enriched in response to roIFNT treatment. Prior to placenta formation, the conceptus relies on the histotrophic secretion from the endometrium into the ULF for nutrients for growth [6]. rbPDI may alter metabolic processes, secretion/import in bEECs to optimise production of metabolites for secretion into the ULF for conceptus nutrition [38].

rbPDI treatment of bEECs also led to the enriched KEGG pathway of “cell-adhesion molecules”, which indicates that PDI may be involved in the process of attachment during the initiation of implantation. All the DEGs in response to rbPDI in bEECs mapped to the KEGG pathway “cell adhesion molecules” were upregulated compared to vehicle control, indicating that PDI secretion by the conceptus would increase adhesion to endometrium.

When looking in detail at the DEGs specific to rbPDI or rbPDI+roIFNT treatment compared to vehicle control, but not those induced by roIFNT [22], the GO terms enriched include cell adhesion molecules, metabolic processes, and other immune-related gene sets. Enriched KEGG pathways from this set of DEGS are all related to the immune response, including: NFKB, TNF, IL-17, and toll-like receptor signalling pathways- which are all pro-inflammatory. Although it seems counterintuitive for the conceptus to secrete PDI, which induces a proinflammatory response as seen in this project, studies have shown increases in natural killer cells [39], macrophages [40], or dendritic cells [41] in the bovine endometrium during pregnancy, which are all immune cells recruited by pro-inflammatory signals. PDI may therefore have a role in recruiting these cells to the endometrium, although more evidence is required to confirm this. Overall, these data further support our hypothesis that PDI may support the maternal immune system modulation elicited by IFNT, and potentially facilitate implantation and/or the histotroph secreted by the endometrium.

In bovine endometrial cells *in vitro,* the number of DEGs induced by rbPDI are fewer in stromal than epithelial cells. This may be due to the *in vivo* endometrial architecture, where stromal cells reside below the epithelial layer which lines the uterine cavity. The epithelial cells are therefore the first contact for embryo-derived factors, and these data indicate that the two cell types may respond differently to embryo-derived proteins *in vivo.* Like bEECs, the response to rbPDI in bESCs was associated with GO terms related to immune function and cell–cell adhesion.

### Human endometrial cells have a limited response to PDI *in vitro*

As described, rbPDI altered the expression of fewer transcripts in human endometrial epithelial cells than in bovine endometrial cells. This could indicate a species-specific response to PDI protein, or that the rbPDI transcript interacts differently with human cells than human PDI. Recently published work demonstrated that human endometrial cells responded to recombinant human PDI (with increased expression of microRNA-324-5p), whereas rbPDI induced a different response in bovine endometrial cells (decreased expression of microRNAs −185-5p, −542-3p, and -151a-3p), supporting a model of species-specific response [21]. The endometrial transcriptional response in Ishikawa human endometrial epithelial cells to rbPDI mostly differed when compared to bEECs, however, the transcript *MNS1* was commonly downregulated in both species in response to rbPDI compared to vehicle control.

*MNS1* encodes the meiosis specific nuclear structural protein (MNS1) which is involved in both cilia and flagella assembly [42]. MNS1 has been implicated to be critical in the process of spermatogenesis [43], and extensively researched in the context of male fertility. In endometrium, a recent study described *MNS1* expression being altered in human endometrial organoids in response to estrogen treatment [44], and in cattle that *MNS1* expression in peripheral white blood cells was significantly different between artificially inseminated compared to natural breeding pregnant heifers [45]. The role and mechanism of action of *MNS1* in the endometrium are not currently known and warrant further investigation.

Finally, this study utilised an immortalised human endometrial epithelial cell line (Ishikawa cells), which was derived from an adenocarcinoma. This cell line could therefore not be representative of all of the function (s) or responsive elements of the human *in vivo* endometrial epithelium, and potentially not respond to PDI as *in vivo* derived cells would. Cancer-derived cell lines are known to undergo genetic drift over time due to rapid proliferation and cancer-driven mechanisms [46]. Despite this limitation, many groups have utilised this cell line to provide valuable insights into endometrial biology in humans and they provide a resource which is easily accessible and doesn’t require ethical considerations [47, 48]. We acknowledge that further studies, across additional species and using different human models and primary human cells, are needed to fully assess conservation of PDIs role in implantation.

### PDI may mediate implantation at the attachment stage via *MNS1*

Although rbPDI treatment of human endometrial Ishikawa cells had no significant effect on BeWo trophoblast cell spheroid attachment in this project, recent work demonstrated that inhibiting PDI significantly increases attachment of Jeg-3 spheroids onto AN3CA human endometrial cells [18]. Interestingly, PDI expression was found to be significantly higher in AN3CA cells compared to Ishikawa cells, and Ishikawa cells are considered to be in a “receptive” state [49] whereas AN3CA cells are not [50]. One possible explanation is that PDI acts to decrease attachment to non-receptive endometrium, acting as a timing mechanism to ensure attachment only to a receptive endometrium or even acting as a mechanism to prevent premature implantation, such as in the fallopian tube. One study supporting this hypothesis demonstrated that PDI expression is significantly higher in the stromal cells of non-receptive endometrium of bonnet monkeys compared to receptive endometrium [51]. Although the study also found no difference in PDI expression in the epithelial glands, the authors do not appear to have assessed the luminal epithelium.

Although implantation attachment wasn’t shown to be altered by rbPDI in this investigation, there are limitations to the work done here. rbPDI may not function in exactly the same way as the human ortholog, and therefore may not act upon the human endometrial Ishikawa cells to produce an effect on attachment. BeWo cells are also an adenocarcinoma cell line derived from trophoblast cells and therefore may not act as benign trophoblast cells would *in vivo*, however this assay have been routinely used to study human embryo implantation attachment in different contexts [52, 53]. Also, only one concentration of rbPDI was tested, whereas influencing trophoblast spheroid attachment may occur at a specific range of concentration or timepoint.

As *MNS1* was commonly downregulated in both human and bEECs in response to rbPDI exposure, we next sought to determine if *MNS1* has a role in the attachment phase of implantation. When *MNS1* expression was decreased by >86% in Ishikawa cells using siRNA knockdown, the rate of BeWo spheroid attachment significantly decreased compared to controls. *MNS1* expression was decreased in response to rbPDI treatment in both human endometrial epithelial Ishikawa cells and bEECs, but only a 50% decrease (log2 fold change −1.009), which could be why we have observed an effect on attachment in only the *MNS1* siRNA knockdown, but not the PDI treatment. Therefore, exposure to PDI may decrease trophoblast attachment to the endometrium, supported by work demonstrating that PDI inhibition increases spheroid attachment [18], and that when *MNS1* expression is reduced (as occurs in PDI exposure) attachment reduces. This work shows that *MNS1* may be a mechanism by which PDI mediates implantation, perhaps in a reproductive cycle timing specific manner. Further work is needed to elucidate if this mechanism is applicable *in vivo* and in other species, and development of *in vitro* implantation assays in species which undergo conceptus elongation (such as bovine) are required to fully understand species-specific mechanisms.

### Recombinant bovine PDI alters the endometrial secretome in a species-specific manner

Collection of the conditioned or “spent” medium from the microfluidics devices seeded with human endometrial epithelial Ishikawa cells or bEECs allowed the secretome of the cells to be analysed by mass spectrometry. Microfluidic-based cell culture was utilised for this project as a tool to provide continual exposure of the endometrial cells to PDI protein. This was to replicate the likely continual secretion of conceptus-derived proteins to which the endometrium is then exposed to, rather than using single or multi-exposure timepoints and potentially not capturing the exposure effects.

Assigning protein IDs to the proteins secreted by human endometrial epithelial Ishikawa cells in response to rbPDI showed that only 2/17 proteins which were differentially abundant were assigned homo sapiens species identifiers. This discrepancy is speculated to be due to the bovine FBS used in the culture medium and/or false positives. The two human differentially abundant proteins, mitochondrial import inner membrane translocase subunit TIM16 and limbic system-associated membrane protein (fragment), were only slightly higher in abundance compared to vehicle control (0.16- and 0.08-log2 fold change respectively) in the conditioned medium. TIM16 was in 12% greater abundance in the conditioned medium after rbPDI treatment than vehicle control, and is involved in ATP-dependent protein translocation into the mitochondrial matrix as a component of the TIM23 complex [54]. TIM23 is involved in the import of approximately 60% of mitochondrial proteins, and alterations in the mitochondrial proteome are associated with changes in growth conditions and are mostly involved in energy metabolism [55]. Therefore, the secretion of TIM16 by endometrial cells in response to PDI could aid the function of the conceptus mitochondria to increase energy availability and growth, and as a result increased viability of the conceptus. The gene transcripts for neither protein were differentially altered (padj<0.05 and log2 fold change >1 or < −1), although the transcript *LSAMP* had a log2fold change 0.64 (padj 0.0164) compared to vehicle control when treated with rbPDI in Ishikawa cells, which is equivalent to a 56% increase in expression. This indicates that PDI may increase the expression and secretion of LSAMP but may only increase the secretion of TIM16.

There were 0 commonly differentially abundant proteins present in the conditioned medium between Ishikawa cells and bEECs when compared to vehicle control samples. Therefore, the response to rbPDI altering the secretome of these cells may be species-specific.

In bEEC conditioned medium serum amyloid A protein was the most highly differentially abundant protein, at 3.72-fold the amount of serum amyloid A present following treatment with rbPDI compared to control. Serum amyloid A is involved in cell–cell communication [56] but was not found in the ULF from pregnant cattle on day 15, 16, or 18 [8, 57], indicating that it may be an artifact of *in vitro* cell culture treatment with rbPDI, or that it is only produced very locally and/or immediately utilised *in vivo*. Tubulin alpha 1D chain has however been identified in pregnant ULF on day 16 from cattle [8, 57, 58] and was increased 0.64-fold during rbPDI treatment. Interestingly, tubulin alpha 1D was also secreted by bovine day 16 conceptuses *in vitro* [58]*.* None of the bovine conditioned medium differentially abundant proteins (Table 2) were differentially expressed in the human endometrial epithelial cells (Supplementary table 20, padj<0.05, log2foldchange >1 or < −1). However, bovine SAA3 protein found to be increased in conditioned medium cultured with bEECs, was also increased in expression in bEECs in static culture. Although the two culture methods used means the data is not directly comparable, this indicates that the secretory response observed in bovine is supported by the transcriptional data.

PDI is a protein with a range of known cellular functions, primarily acting in the endoplasmic reticulum and at the cell membrane [16], and is known to be expressed in human endometrial cells. Forde *et al* did not find PDI in the ULF of cyclic heifers, only in pregnant heifers [8]. Although there is likely an undetectable level of PDI present in ULF, and expression in endometrial cells, secretion of PDI by the conceptus would cause an increase in local concentrations of PDI and we have begun to elucidate the potential consequences of conceptus-derived PDI acting upon the endometrium. PDI was present in the conditioned medium from the vehicle control samples from both bovine and human, however this may be from the FBS used in culture, which could be influencing our results. To our knowledge, PDI has not been evidenced to be secreted from human endometrial cells, *in vivo* or *in vitro*. This study is limited to the concentration and length of rbPDI treatment utilised (1000 ng/ml and 24 h). As many *in vitro* studies use 1000 ng/ml IFNT to study the effects, we limited this study to 1000 ng/ml as PDI had a lower abundance in conditioned medium and ULF than IFNT [8], however we acknowledge that PDI may have a greater or wholly different effect at different concentrations and treatment length. Finally, the use of primary human endometrial cells in this system would provide greater insight into the human endometrial secretome, as cancerous cell lines do not fully recapitulate the *in vivo* endometrium as discussed above.

### Summary

In conclusion we have demonstrated that PDI, a conceptus-derived protein, with highly conserved amino acid sequence across placental mammals alters the transcriptome of the endometrial epithelium in a species-specific manner. In bovine PDI enhances the IFNT simulated response during the peri-implantation period of pregnancy, as well as modifying PDI-specific transcripts in the endometrial epithelial and stromal cells. One transcript, *MNS1* is modified by PDI in two species with divergent implantation strategies i.e., human and bovine, and modifies the ability of BeWo spheroids to attach. Exposure of epithelial cells to PDI also modified the secretome in a species-specific manner. Collectively these data demonstrate that PDI plays a species-specific role in endometrial function in mammals with different implantation strategies.

## Supplementary Material

PDI_paper_Supplementary_tables_ioaf263

Tinning_et_al_PDI_Supplementary_methods_ioaf263

## Data Availability

All transcriptomic data is available on GEO (GSE308141). The mass spectrometry proteomics data have been deposited to the ProteomeXchange Consortium via the PRIDE [59] partner repository with the dataset identifier PXD067948.
