# Supplementary material for: The embryo-derived protein PDI is highly conserved among placental mammals and alters the endometrial transcriptome and secretome in vitro across species with differing implantation strategies
Source: Biol Reprod. 2025 Dec 4;114(4):1295–311. doi: 10.1093/biolre/ioaf263 (PMC13079457; doi:10.1093/biolre/ioaf263)
Supplement: Tinning_et_al_PDI_Supplementary_methods_ioaf263 [file tinning_et_al_pdi_supplementary_methods_ioaf263.docx]

#### **Supplementary methods**

Supplementary methods table 1. qRT-PCR primers designed to target bovine gene sequences specific to endometrial receptivity. Primers were designed using Primer Blast software (<https://www.ncbi.nlm.nih.gov/tools/primer-blast/>).

| Gene | Accession number | Forward sequence (5’-3’) | Reverse sequence (3’-5’) | Product length (bp) |
| --- | --- | --- | --- | --- |
| *DKK1* | NM_001205544.1 | CCATTGACAACCACCAGCCGTATC | CCGACAGGCGAGGCAGATTT | 113 |
| *MX1* | NM_173940.2 | TCCATCCGACTACATTTCCA | CTGCCTCCTTCTCTCTGACC | 94 |
| *MX2* | NM_173941.2 | GGGCAGCGGAATCATCACCC | AGCTGCTGCGTAATGTTGCGGTA | 102 |
| *ISG15* | NM_174366.1 | TGACGGTGAAGATGCTAGGG | ACTGCTTCAGCTCGGATACC | 78 |
| *TKT* | NM_001003906.1 | GGCCGATCAGATCATCCAGG | GCGGGTAGCTATCTTGTCCC | 157 |
| *FABP3* | NM_174313.2 | GTGCGGGAAATGGTTGAC | GTAGCCCACTGGCAGAAGAG | 131 |
| *RSAD2* | NM_001045941.1 | GGTCTGCTGATGCTGAAAGA | GCTCCTGCTTACAGAACTTC | 118 |
| *GAPDH* | NM_001034034.2 | TGAACACTGGAGAAACC | CGCCTGCTTCACCACCTT | 57 |
| *ACTB* | NM_173979.3 | TGGATGATGATATTGC | AAGCCGGCCTTGCACAT | 61 |

Supplementary methods table 2. qRT-PCR primers designed to target human gene sequences specific to PDI. Primers were designed using Primer Blast software (<https://www.ncbi.nlm.nih.gov/tools/primer-blast/>).

| Gene | Accession number | Forward sequence (5’-3’) | Reverse sequence (3’-5’) | Product length (bp) |
| --- | --- | --- | --- | --- |
| *MTTP* | NM_001205544.1 | GTGTTTTGGGAGTGGGAGGT | GGAGTGACCCTCTTCAGAACAAGA | 113 |
| *P4HA3* | NM_173940.2 | GGCACTGTTTTGGTGGAACC | ACTTGTTGGCCACCCACTTA | 94 |
| *P4HA1* | NM_173941.2 | TACAACCTCTTGCGGAGCGG | TTGGAGACCCACTTGCACC | 102 |
| *ERO1L* | NM_174366.1 | CAAAACCAAGGCTGAGCCGA | TTTCATAGCCAGAGAGCCAGG | 78 |
| *HSPA5* | NM_001003906.1 | GAACGTCTGATTGGCGATGC | TCAACCACCTTGAACGGCAA | 157 |
| *HSP90B1* | NM_174313.2 | GCCCAAAGTCACCGTCAAGG | GCCAAATCCTTTCTCTCCTGTAGC | 131 |
| *PPIB* | NM_001045941.1 | AGCAGAACATCGACTGTGGG | CCACAGATGTCGGGACCAAA | 118 |
| *CALR* | NM_001034034.2 | CAGAGGCAAGCCGACTCAAA | CACCCCAGACTTGGTCTCAC | 57 |
| *OS9* | NM_173979.3 | AGAAAATCTGGCACCACACC | TAGCACAGCCTGGATAGCAA | 61 |
| *ACTB* | NM_001101.5 | TTTCTATAAATTGAGCCCGCAGC | TGGCTCGGCTGGCGC | 173 |
| *PPIA* | NM_021130.5 | GCCGAGGAAAACCGTGTACT | GTCTGCAAACAGCTCAAAGGA | 108 |

#### **Implantation Assay Transfection validation**

Following optimisation of transfection efficiency (Supplementary figure S1), *MNS1* knockdown was validated by plating Ishikawa cells (ECACC 99040201, passage 31) at a density of 100,000 cells per well in 12 well plates in triplicate (n=3) in 1mL complete human medium. After 24 hr the medium was aspirated, 1mL PBS added to wash cells and aspirated, and replenished with 800μL antibiotic-free human medium. A siRNA solution was made up for the *MNS1* siRNA (Horizon Discovery, UK) and the non-targeting siRNA (Horizon Discovery, UK) (1.4μL 100μM siRNA + 350μL OptiMEM). A lipofectamine solution was also prepared (900μL OptiMEM [Gibco, Massachusetts, US] + 18μL Lipofectamine 2000 [Invitrogen, St. Louis, US]). Following a 20-minute incubation together, the following solutions were added to each corresponding well: 1) Control (200μL OptiMEM only), 2) Vehicle control (100μL OptiMEM + 100μL Lipofectamine solution), 3) Non-targeting siRNA (100μL Lipofectamine solution + 100μL siRNA solution), 4) *MNS1* siRNA (100μL Lipofectamine solution + 100μL siRNA solution). After 4 hours incubation (37°C/5% CO_2_), medium was aspirated from the wells and replaced with complete human medium. Twenty-four hours after transfection, medium was aspirated, 1mL PBS added and aspirated to wash, and 700μL Qiazol (Qiagen, Germany) added to lyse cells. Cell lysate was collected in a 1.5mL Eppendorf, snap-frozen, and stored at -80°C until RNA extraction. RNA was extracted using the Qiagen miRNeasy Mini kit (Qiagen, Germany) with on-column DNA digestion (RNase-Free DNase set [Qiagen, Germany]) as per the manufacturers protocol and eluted in 50μL ultra-pure distilled water (Invitrogen, St. Louis, US). Purity and concentration of RNA was analysed using a NanoDrop ND-1000 Spectrophotometer (LabTech International, UK). RNA concentration was diluted to 200 ng/mL in ultra-pure distilled water (Invitrogen, St. Louis, US). Ten μL RNA was reverse transcribed into cDNA using the high-capacity cDNA synthesis kit (Applied Biosystems, Massachusetts, US) as described above. Two μL cDNA was plated into a 384 well clear plates (Bio-Rab Laboratories, California, US), in duplicate, per sample. Eight μL master mix, containing 0.25μL forward and reverse primers (IDT, Iowa, US), 2.5μ ultra-pure distilled water (Invitrogen, St. Louis, US), and 5μL SYBR Green (Roche, Switzerland) per well, was added. The plate was sealed with a microseal PCR plate sealing film (Bio-Rab Laboratories, California, US) and centrifuged briefly. The plate was then run using the standard Roche Lightcycler 480 mRNA programme with 40 cycles. Ct values were calculated using the Roche Lightcycler 480 software. The 2^-ΔΔCt^ method using *ACTB* as a normaliser gene, comparing all samples to the non-targeting samples, was used to determine fold change difference in expression. % knockdown of *MNS1* was calculated by taking the geometric mean of the 2^-ΔΔCt^ result and using the following equation: % knockdown = 100 - (100 * geomean of 2^-ΔΔCt^ result).

#### **Microfluidics and conditioned medium proteomics**

#### Human endometrial epithelial cells

On day 1 of the experiment, human endometrial Ishikawa cells (passage 20, ECACC 99040201, n=3) were seeded at a density of 1,000,000/mL into IbiFlow 0.4 channel slides (Ibidi, Germany) (approximately 60μL to fill channel) in complete human medium, with the inlet and outlet marked. Cells were left to adhere for 4 hours in the incubator (37°C/5% CO_2_) before 100μL complete human medium was added to the inlet and the device returned to the incubator. Medium was replenished daily until day 4.

On day 3 the treatments were prepared in triplicate and put into 5mL leur lock syringes (Terumo, Japan) and placed into the incubator (37°C/5% CO_2_) overnight until attaching to devices as described below on day 4. Vehicle control treatment was 4.95mL complete human medium with 10% exosome depleted FBS + 50μL PBS. rbPDI treatment was 4.95mL complete human medium with 10% exosome depleted FBS + 50μL rbPDI (100 μg/mL).

On day 4, medium was removed from the outlet, and the channel washed three times with 100μL PBS (37°C) added to the inlet and removed gently from the outlet. Complete human medium with 10% exosome depleted FBS (Gibco, Massachusetts, US) was replenished and device placed into incubator. The syringes were removed from the incubator, flicked to remove all bubbles, and placed securely into a NE-1600 syringe pump (New Era Pump Systems, New York, US) set up on an incubator shelf in a laminar flow hood. Each syringe had an Ibidi luer lock connector female (Ibidi, Germany) attached with approximately 10cm 0.8mm ID sterile silicone tubing (Ibidi, Germany). To the other end of the tubing an Ibidi elbow leur connector (Ibidi, Germany) was attached and media gently pushed through until no visible bubbles remained and a droplet of medium was present at the outlet of the elbow leur connector.

The device containing the cells was removed from the incubator and PBS added to the inlet until full. The droplet on the elbow connector was then connected to the full droplet of PBS in the device inlet and pressed down firmly at a 90-degree angle, then straightened to seal tightly, and repeated for all channels and syringes, as seen in Figures 1 and 2.

Six 7mL bijous were prepared by pushing a hole in the lid. A second piece of 10cm sterile silicone 0.8mm ID tubing was connected to an elbow leur connector. A 1mL syringe (BD Plastipak, Michigan, US) with blunt needle (Sol-Millennium, Illionois, US) was used to fill the 10cm piece of tubing with PBS until a droplet of PBS emerged from the elbow leur connector. With the syringe still attached, the droplet of PBS on the elbow leur connector was joined with the droplet of PBS filling the outlet of the device channels and repeated for all six channels. The elbow leur connector was pressed firmly down into the outlet of the device at a 90-degree angle, whilst simultaneously removing the blunt needle. The elbow leur connector was then straightened to tightly connect with a firm seal. The end of the tubing was then placed through the hole into the 7mL bijou to collect the conditioned medium. The whole system was then taped securely with masking tape, and the shelf returned to the incubator (37°C/5% CO_2_). The system used is shown in Figures 1 and 2.

The syringe pump was switched on, set to a syringe internal diameter of 13mm, and a flow rate of 1uL/min, and set to run for 24 hours. This flow rate was selected because uterine tubal secretions was found to be 1.43mL/24 hours during dioestrus and 1.54mL/24 hours during oestrus in cattle (33), with the rate of 1uL/min equating to 1.44mL/24 hours. After 24 hours, the whole shelf was removed and placed into the laminar flow hood. The conditioned medium was collected from the 7mL bijous and transferred to 2mL sterile Eppendorf tubes and placed into the fridge until they could be processed (which occurred within 1 hour of ending flow). The Ibidi luer lock connector female was uncoupled from the syringes gently and the elbow connectors uncoupled from the devices. The device was then flushed through with PBS three times and then 0.025% trypsin added and placed into the incubator for 3 minutes. After three minutes the device was gently tapped to dislodge the cells and PBS added to the inlet to push the cells to the outlet where they were collected and transferred to a 1.5mL sterile Eppendorf. Cells were pelleted at 500xg for 5 minutes, re-suspended in 1mL PBS, and pelleted at 500xg for 5 minutes. PBS was aspirated and the pellet snap-frozen in liquid nitrogen before transferring to -80°C freezer.

#### Bovine endometrial epithelial cells

bEECs (n=3) were isolated as described above and seeded into devices after 27 days in culture at a density of 500,000 cells/mL in approximately 60μL complete bovine medium per channel. Cells were allowed to adhere for 4 hours in the incubator (37°C/5% CO_2_) and then 100μL complete bovine medium added to the inlet. Medium was replenished daily until day 4. On day 3 the treatments were prepared in triplicate and put into 5mL leur-lock syringes (Terumo, Japan) and placed into the incubator (37°C/5% CO_2_) overnight until attaching to devices. Vehicle control treatment was 4.95mL complete bovine medium with 10% charcoal stripped exosome depleted FBS + 50μL PBS. rbPDI treatment was 4.95mL complete bovine medium with 10% charcoal stripped exosome depleted FBS + 50μL rbPDI (100 μg/mL). On day 4, medium was removed from the outlet and the channel washed three times with 100μL PBS (37°C) added to the inlet and removed gently from the outlet. Complete bovine medium with 10% charcoal stripped exosome depleted FBS (Gibco, Massachusetts, US) was replenished and device placed into incubator. The devices were then attached to the syringes containing treatment in the pump system, and the experiment carried out under the same conditions and samples collected as described in for human microfluidic samples.

#### Conditioned medium mass spectrometry and data analysis

The conditioned medium for both the human and bovine samples were processed from the fridge after collecting the cell pellets, first by centrifuging at 500xg for 10 minutes to remove cells, 2000xg for 10 minutes to remove cell debris, and finally microvesicles (MVs) removed at 14,500xg 4°C for 30 minutes. The supernatant conditioned medium (containing smaller extracellular vesicles) was snap-frozen in liquid nitrogen and transferred to the -80°C freezer.

Following processing, the conditioned medium from both the human and bovine microfluidics were sent to Bristol Proteomics Facility for mass spectrometry analysis. Samples were depleted of bovine albumin using an albumin depletion kit, according to the manufacturer’s protocol (Thermo Fisher Scientific, UK). An equal volume of each depleted sample (equivalent to 30-40µg protein for the human samples, equivalent to 40-50µg protein for the bovine samples) was then digested with trypsin (1.25µg trypsin; 37°C, overnight), labelled with Tandem Mass Tag (TMT) ten plex reagents according to the manufacturer’s protocol (Thermo Fisher Scientific, UK) and the labelled samples pooled.

Pooled samples were desalted using a SepPak cartridge according to the manufacturer’s instructions (Waters, Massachusetts, USA). Eluate from the SepPak cartridge was evaporated to dryness and resuspended in buffer A (20 mM ammonium hydroxide, pH 10) prior to fractionation by high pH reversed-phase chromatography using an Ultimate 3000 liquid chromatography system (Thermo Fisher Scientific, UK). In brief, the sample was loaded onto an XBridge BEH C18 Column (130Å, 3.5 µm, 2.1 mm X 150 mm, Waters, UK) in buffer A and peptides eluted with an increasing gradient of buffer B (20 mM Ammonium Hydroxide in acetonitrile, pH 10) from 0-95% over 60 minutes. The resulting fractions (15 in total) were evaporated to dryness and resuspended in 1% formic acid prior to analysis by nano-LC MSMS using an Orbitrap Fusion Lumos mass spectrometer (Thermo Scientific).

High pH RP fractions were further fractionated using an Ultimate 3000 nano-LC system in line with an Orbitrap Fusion Lumos mass spectrometer (Thermo Scientific). In brief, peptides in 1% (vol/vol) formic acid were injected onto an Acclaim PepMap C18 nano-trap column (Thermo Scientific). After washing with 0.5% (vol/vol) acetonitrile 0.1% (vol/vol) formic acid peptides were resolved on a 250 mm × 75 μm Acclaim PepMap C18 reverse phase analytical column (Thermo Scientific) over a 150 minutes organic gradient, using 7 gradient segments (1-6% solvent B over 1min., 6-15% B over 58min., 15-32%B over 58min., 32-40%B over 5min., 40-90%B over 1min., held at 90%B for 6 minutes and then reduced to 1%B over 1min.) with a flow rate of 300 nl min^−1^. Solvent A was 0.1% formic acid and solvent B was aqueous 80% acetonitrile in 0.1% formic acid. Peptides were ionized by nano-electrospray ionization at 2.0kV using a stainless-steel emitter with an internal diameter of 30 μm (Thermo Scientific) and a capillary temperature of 300°C.

All spectra were acquired using an Orbitrap Fusion Lumos mass spectrometer controlled by Xcalibur 3.0 software (Thermo Scientific) and operated in data-dependent acquisition mode using an SPS-MS3 workflow. FTMS1 spectra were collected at a resolution of 120 000, with an automatic gain control (AGC) target of 200 000 and a max injection time of 50ms. Precursors were filtered with an intensity threshold of 5000, according to charge state (to include charge states 2-7) and with monoisotopic peak determination set to Peptide. Previously interrogated precursors were excluded using a dynamic window (60s +/-10ppm). The MS2 precursors were isolated with a quadrupole isolation window of 0.7m/z. ITMS2 spectra were collected with an AGC target of 10 000, max injection time of 70ms and CID collision energy of 35%.

For FTMS3 analysis, the Orbitrap was operated at 50 000 resolution with an AGC target of 50 000 and a max injection time of 105ms. Precursors were fragmented by high energy collision dissociation (HCD) at a normalised collision energy of 60% to ensure maximal TMT reporter ion yield. Synchronous Precursor Selection (SPS) was enabled to include up to 10 MS2 fragment ions in the FTMS3 scan.

The raw data files were processed and quantified using Proteome Discoverer software v2.1 (Thermo Scientific) and searched against the UniProt Bos taurus database (downloaded September 2020: 46224 entries) and the Uniprot Homo sapiens database (downloaded January 2021: 169297 entries) using the SEQUEST HT algorithm for the human samples. The raw data files were processed and quantified using Proteome Discoverer software v2.1 (Thermo Scientific) and searched against the UniProt Bos taurus database (downloaded October 2021: 37512 entries) and the Uniprot Homo sapiens database (downloaded January 2021: 169297 entries using the SEQUEST HT algorithm for the bovine samples. Peptide precursor mass tolerance was set at 10ppm, and MS/MS tolerance was set at 0.6Da. Search criteria included oxidation of methionine (+15.995Da), acetylation of the protein N-terminus (+42.011Da) and Methionine loss plus acetylation of the protein N-terminus (-89.03Da) as variable modifications and carbamidomethylation of cysteine (+57.021Da) and the addition of the TMT mass tag (+229.163Da) to peptide N-termini and lysine as fixed modifications. Searches were performed with full tryptic digestion and a maximum of 2 missed cleavages were allowed. The reverse database search option was enabled, and all data was filtered to satisfy false discovery rate (FDR) of 5%.

**Supplementary methods figure 1.** siRNA knockdown efficiency *MNS1* compared to non-targeting siRNA control (NT). OptiMEM only media control, lipofectamine vehicle control (VC), non-targeting siRNA (NT), and siRNA targeting *MNS1* treated Ishikawa cells (n=2) for 48 hours. *MNS1* expression determined by qRT-PCR and % knockdown calculated from the 2^-ΔΔCt^ values using *ACTB* as a normaliser gene relative to the non-targeting siRNA treated samples. Figure created in Graphpad Prism.
